# Structured panendoscopy reports improve report completeness and documentation time

**DOI:** 10.1038/s41598-025-27738-8

**Published:** 2025-11-25

**Authors:** Friederike Bärhold, Stephan Matthias Wolpert, Benjamin Philipp Ernst, Georg Long Fei Potthast, Thomas Breuer, Sven Becker, Lotte Pummerer, Martin Holderried

**Affiliations:** 1 Department of Otorhinolaryngology – Head & Neck Surgery, University Hospital Tübingen, Tübingen, Germany; 2 Department of Otorhinolaryngology, University Hospital Frankfurt, Frankfurt, Germany; 3 Department of Otorhinolaryngology, LKH Hochsteiermark State Hospital, Loeben, Austria; 4 Hals-Nasen-Ohren-Center Luzern AG, Luzern, Switzerland; 5 University of Bremen, Bremen, Germany; 6 Department of Medical Strategy, Process and Quality Management, University Hospital Tübingen, Tübingen, Germany; 7 Institute of Health Care and Public Management, University of Hohenheim, 70599 Stuttgart, Germany

**Keywords:** Panendoscopy, Structured reporting, Otorhinolaryngology, Digitalization, Free text reports, Cancer screening, Cancer

## Abstract

Even today, surgical reports are usually dictated in a free text form (FTR), leading to a wide range in report-quality. This study investigated the use of a fully structured panendoscopy report (SR) compared to FTRs. 64 panendoscopies were performed by three experienced head and neck surgeons. The surgical reports were created as both FTRs and SRs, which were examined regarding time to completion and content using a multilevel regression analysis. User satisfaction was evaluated using a questionnaire. There was no significant difference in time to complete the SRs compared to FTRs. The completeness ratings of SRs were significantly higher than for FTRs (81% vs. 66%, *p* < 0.001), leading to increased report quality. Overall user satisfaction was higher for SRs than for conventional FTRs (VAS 8.1 vs. 3.5, *p* < 0.001). The SRs proved to be fast to complete and more comprehensive with a higher completeness of content. Participating surgeons indicated that they preferred SRs over FTRs because of their advantages in terms of structure, guidance for inexperienced residents and non-native speakers. The data stratification also enables secondary data use to further develop deep learning algorithms in patient care and research.

## Introduction

 Panendoscopies are frequently conducted procedures in head and neck surgery that assess the mucosa of the entire upper respiratory tract, trachea, and esophagus under general anesthesia. They are particularly important in head and neck oncology for diagnosis and staging of suspected tumors. Therefore, the panendoscopy report plays a crucial role for therapy planning and the evaluation of the different treatment options^1^. It is essential that the report contains detailed information about the scope of the diagnostic procedure, the pathological findings, the size and extent of the tumor, and the surgeon’s assessment regarding the tumor’s resectability. Incomplete or inaccurate documentation can lead to misinterpretation of tumor extent, resulting in misdiagnosis of the tumor stage and finally leading to inappropriate treatment with increased risks and potential worsening of prognosis for the patient.

Structured reports (SR) have emerged as an alternative to conventional free text reports in various fields to ensure an adequate readability, accuracy and completeness of medical documentation. Especially in the fields of radiology and pathology, several software solutions and structured reporting templates are available, usually related to a specific diagnostic modality and one single body part^2–8^. Furthermore, the use of SRs has been shown to lead to improved readability and completeness, high interrater reliability, and facilitated learning progress for medical students^9,10^. Regarding the time to complete the reports, the previously published results are inconclusive. Some authors reported time savings^11,12^, while others reported no change or increased time when introducing SRs^3,13,14^. In cases where the time required for documentation increased, participants criticized too many restrictions and regarded the template structure as too complicated, resulting in a reduced usability of the SR template^13^. Publications analyzing the most common errors in medical documentation concluded that the error rate in SR was lower compared to FTR^15,16^.

Although significant progress has recently been made in the field of artificial intelligence (AI) and machine learning (ML) in medicine^17^, including tools for image recognition and enhancement^18,19^, diagnostics, and decision support^20^, the integration of large language models for clinical documentation remains a challenge. Regulatory concerns, limited transparency, and the risk of incorrect or inconsistent output currently restrict their use in critical medical documentation^21,22^. In contrast, structured reporting itself does not yet use deep learning algorithms, but rather predefined, reproducible, and transparent results that depend entirely on the input options and are therefore completely traceable and verifiable. Although AI is not directly applied in this study, the structured, machine-readable documentation examined in this study forms an essential basis for future AI applications in medicine.

Currently, there are still different forms of ‘structured reporting’ in everyday clinical practice. Most hospitals use partially structured templates, both digitally and on paper. These usually include a pre-defined general pattern, but also individual free text elements. However, the full potential of structured reporting can only be realized if all information is captured via a digital interface that enables machine-readable and interoperable storage of the input for automated data analysis^2,23^. This allows the automatic triggering of downstream processes related to patient safety^24–26^.

Even though the first structured reports for routine clinical care, e.g. structured neurotological reporting or structured documentation of biologic treatment in Chronic Rhinosinusitis with Nasal Polyps are in use^27,28^, there has been no development of a structured operation report in the field of Head and Neck Surgery so far.

The scope of this study was to assess the usability of a newly developed fully structured documentation tool for panendoscopy reports with automatically generated, fully semantic content.

## Methods

In this single-center prospective study, a SR template was developed to create fully structured panendoscopy reports. Subsequently, a total of 64 panendoscopies were performed by three experienced head and neck surgeons with 16 to 22 years of surgical experience and between 1 and 3 panendoscopies per week at the University Department of Otolaryngology, Head and Neck Surgery, University Hospital Tübingen, Germany. The corresponding reports were created as FTRs and SRs over the course of two years. After completion of the panendoscopy procedure, the routine FTR report was first dictated by the surgeon. After routine reporting a SR was created by the same head and neck surgeon using the previously developed digitally and semantically structured report form. The SRs and FTRs were examined in terms of completeness of content, time needed to completion, and user satisfaction. The study was approved by the ethics committee of the medical faculty of the Eberhard-Karls-University and the University Hospital Tübingen, Germany (project number 761/2024). Informed consent was obtained by all participating patients. All methods were carried out in accordance with the Declaration of Helsinki and guidelines of Good Clinical Practice.

### Calculation of sample size

The required sample size for the comparison of the content quality of free-text and fully digitally structured surgical reports was calculated based on the expected effect size. Since a large effect for the completeness of the content of SRs compared to FTRs has already been reported in previous studies^3,8^, an effect size of 0.8 was assumed for the sample size calculation. The power was set at 80% and the significance level was set at α = 0.05. Using these parameters, the minimum number of patients to assess the report content was determined, which resulted in *n* = 25^29^. Due to the high complexity of the panendoscopy report compared to the used reports in previous studies on SR in the field of medical imaging and the inconsistent data in the literature regarding the comparative time requirements for SR and FTR^3,13,14,30^, a larger sample size was aimed for. With about 240 panendoscopies per year, recruiting 64 patients over 2 years was considered realistic.

### FTR and SR

The FTRs were generated using dictation, which was the department’s standard method to create operation reports. The audio file was then sent to the transcription office via the department’s dictation software. After transcription, the written text was corrected if necessary, and finally approved by the respective physician. Until the time of this study, no digital data entry mask was used. Some of the physicians used similar reports from previous operations as a reference or even used predefined text modules that were then individualized. This approach was applied on an individual basis without transparent standardization by the hospital`s medical management team.

**Fig. 1:**
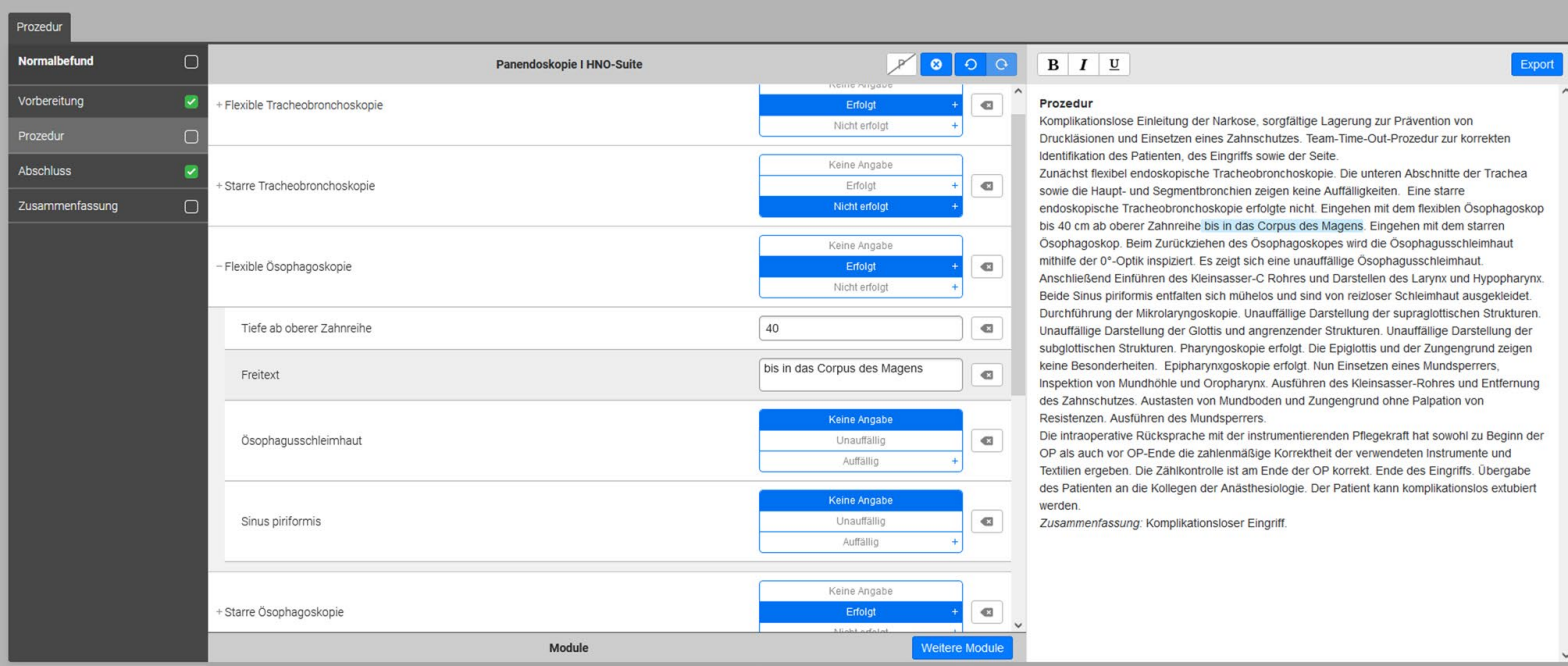
This is a screenshot of the web-based tool developed by Smart Reporting GmbH, Munich, Germany (https://www.smart-reporting.com*).* The panendoscopy report template shown was created by a group of experienced head and neck surgeons prior to the study. On the left, a side bar provides a general structure; a standard panendoscopy report can be generated by clicking on ‘normal findings’ (‘Normalbefund’). In the middle, a clickable decision tree guides the surgeon through the entire operation. The automatically generated full text is shown on the right.

**Fig. 2:**
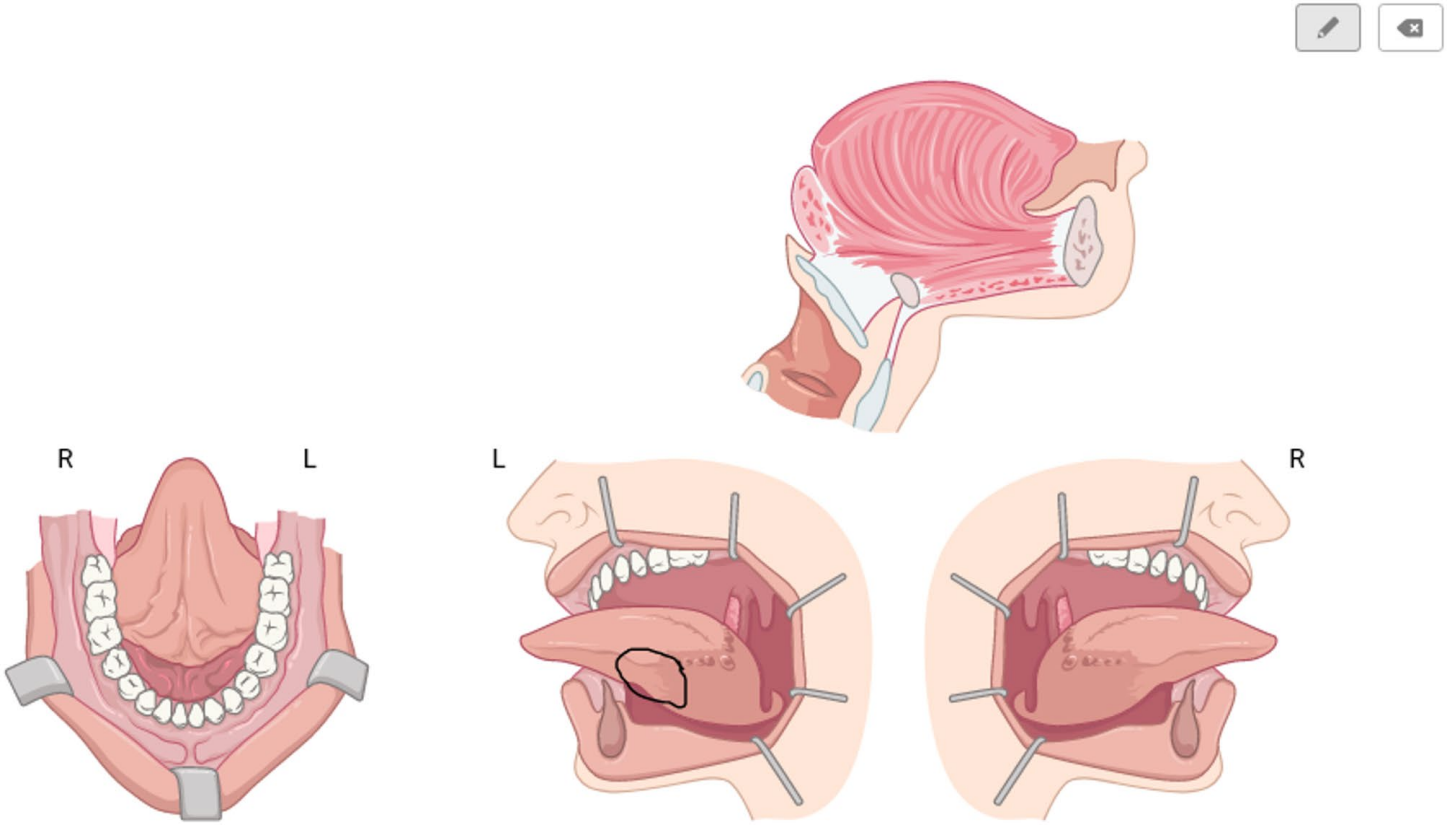
The structured template used in this study includes sketch elements that allow the surgeon to draw in pathological findings. This graphical presentation of information can be a valuable addition to the written report.

For the implementation of the SRs, a web-based software (Smart Reporting GmbH, Munich, Germany, https://www.smart-reporting.com/de/) was used to create a specific template for the structured reporting of panendoscopies. The template was developed in collaboration with highly experienced otorhinolaryngologists with proficiency in head and neck surgery, quality management, and health-IT. The template was structured according to the standard surgical procedure of the Department of Otolaryngology, Head and Neck Surgery, University Hospital Tübingen, Germany where the study was conducted, and is based on the current guidelines of the relevant medical societies^31,32^. It thus guides the surgeons through all steps of the panendoscopy reporting-procedure. The user interface provides clickable decision trees (see Fig. 1) including graphical elements to specify the localization and extent of the pathologies detected during the surgical procedure (see Fig. 2). The software generates full text reports from the selected predefined text phrases. Free text elements and additional comments can be added at any point in the report to cover individual aspects.

### Evaluation of the reports

The criteria for the evaluation of the reports were time required to complete the documentation, the completeness of the reports, and user satisfaction. Three highly experienced senior physicians from the Department of Otorhinolaryngology – Head & Neck Surgery at the University Hospital of Tübingen, Germany, each with between 17 and 22 years of practical professional experience in the medical field were asked to document the panendoscopies they performed themselves in their usual manner as FTR. In a second step, these physicians documented the procedure using SR. The time required for each documentation method was measured. The time required by the transcription office for the transcription of the audio file and the subsequent correction by the respective physician, which was required for FTR, was also included.

User satisfaction was evaluated using a questionnaire with several items on a visual analog scale (VAS) in terms of overall satisfaction, structure, usability, readability, content and potential benefit for inexperienced physicians. The questionnaire was based on the publication of Ernst et al.^3^, which was used to evaluate a structured ultrasound template and was validated and published in 2019. In addition, the professional experience and experience with digital applications of the physicians were assessed.

The content of the reports was evaluated based on overall completeness (i.e. induction of anaesthesia, reporting on tracheobronchoscopy, oesophagoscopy, microlaryngoscopy, epipharyngoscopy, and overall summary assessment). The completeness of the report was scored and compared to the FTR. One point was awarded for each relevant information mentioned in the report, with a maximum of 32 points. The results are given as a percentage of the maximum possible score (insufficient: 0–20%, poor: 20–40%, moderate: 40–60%, high: 60–80%, very high: 80–100%).

### Statistical analysis

Data are presented as the mean ± standard deviation. A p-value of less than 0,05 was considered statistically significant at *α* = 5%. Time to complete the documentation was measured in seconds. A multilevel regression model was conducted. The subjective assessment of SR- and FTR-usability was measured using a VAS (min. 0, max. 10) and the results are presented as means. All statistical analyses were performed with Microsoft Excel 2019 (Microsoft Corporation, Redmond, Seattle, USA) and R Statistical Software (v4.3.2; R Core Team 2021)^33^, using the package Ime4^34^.

## Results

A total of 64 patients were included in the study. All 128 reports (*n* = 64 for FTR and *n* = 64 for SR) were examined for the time to completion. 64 reports (*n* = 32 for FTR and 32 for SR) were analyzed for completeness of content. The patient-specific variables of sex, reason for the panendoscopy, tumor localization, number of tissue samples taken, and the histopathological results of the tissue samples taken are shown in detail in Table 1.

**Table 1:** Patient characteristics of the study cohort including demographic data and specific information on the panendoscopies are listed in this table. The absolute number of patients is given with the corresponding percentage of the study population in brackets.

Demographic Data	*n* (%)	
*Number of Patients*	64	
*Age at surgery (mean ± SD)*	65 ± 10.9 years	
*Sex*	male	55 (85.9)
	female	9 (14.1)
*Reason for surgery*	proven tumor	18 (28.1)
	suspected tumor	44 (68.8)
	dysphagia	1 (1.6)
	leukoplakia	3 (4.7)
*Localization of the lesion or tumor*	Epipharynx	1 (1.6)
	Oropharynx	18 (28.1)
	Hypopharynx	10 (15.6)
	Larynx	12 (18.8)
	oral cavity	19 (29.7)
	Esophagus	1 (1.6)
	CUP_1_	2 (3.1)
*Number of samples*	0	11 (17.2)
	1	28 (43.8)
	2	11 (17.2)
	3	5 (7.8)
	4	1 (1.6)
	5	1 (1.6)
*Histopathological findings*	SCC	47 (73.4)
	CIS_2_, SIN_3_ II/III	4 (6.3)
	Lymphoma	3 (4.7)
	no malignancy	10 (6.4)

^1^cancer of unknown primary, ^2^carcinoma in situ, ^3^squamous intraepithelial neoplasia.

### Report analysis

**Fig. 3:**
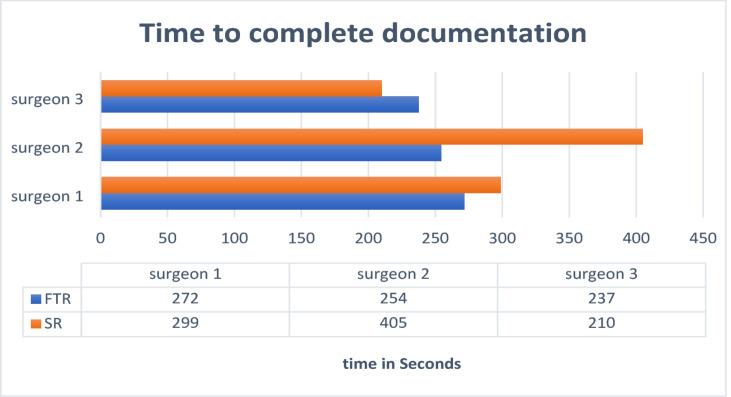
This graph shows the time taken to complete the documentation separately for the three different surgeons using the structured report form (SR; orange) and free text (FTR; blue). A data table shows the exact time in seconds for SR and FTR.

The multilevel regression analyses showed that there was no significant difference in time taken by the surgeons to create the FTRs and SRs: Dictating and correcting the FTR dictation (*mean value* = 259.48 ± 103.21 s) took a similar amount of time as entering the SR into the digital template using the decision tree (*mean value* = 251.16 ± 57.93, *p* = 0.536). The time taken by the transcription office to transcribe the dictation for the FTRs was also measured and amounted to an additional 352.4 ± 100 s. Taking into account this transcription time, the total time for creating the FTRs was significantly longer than the time required to create the SRs (mean value 587 s vs. 259 s, *p* < 0.001). The detailed results are shown in Fig. 3.

**Fig. 4:**
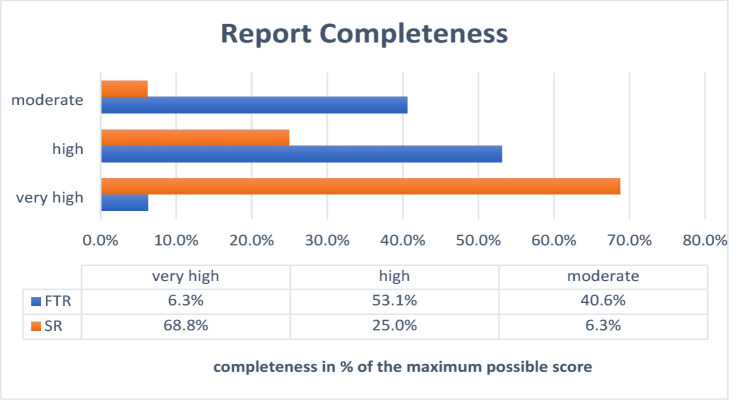
The completeness of documentation is given in this graph for structured reports (SR; orange) and free text reports (FTR; blue) separately, grouped as follows: insufficient: 0–20%, poor: 20–40%, moderate: 40–60%, high: 60–80%, very high: 80–100% completeness. No report achieved less than 40% completeness. The data table below shows the exact percentages of panendoscopy reports in each category.

The completeness of the panendoscopy reports was significantly higher for the SRs (*mean* value = 81.84 ± 0.11%) than for the panendoscopy reports generated using free text (*mean* value = 66.03 ± 0.09%) (*p* < 0.001). 68.8% of the SRs achieved a very high level of completeness, compared to 6.3% of FTRs. None of the reports were poor or insufficient, regardless of the documentation method. There was no correlation between the duration of documentation and the completeness of the content (R² = 0.02; *p* = 0.246). A detailed analysis of the reports is shown in Fig. 4.

### User satisfaction

**Fig. 5:**
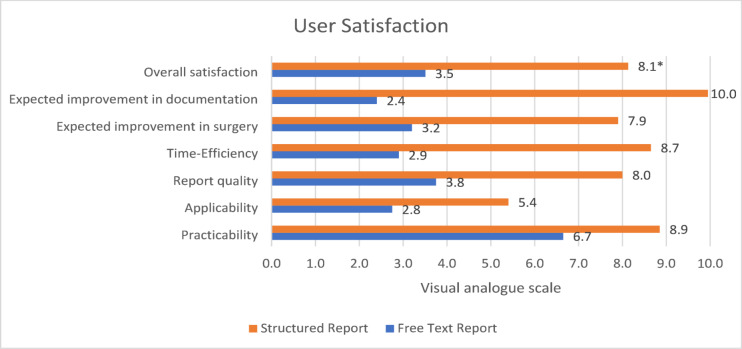
User satisfaction for the two documentation methods structured reporting (orange) and free text reporting (blue) on a VAS (scores 0–10) are given as the mean. The items surveyed were the expected benefit for inexperienced physicians to perform surgery and to improve documentation, time efficiency, report quality, applicability and practicability. In all categories, SRs were superior to FTRs with overall satisfaction being significantly higher regarding SRs (*p < *0.001*).

The evaluation of the VAS questionnaire confirmed a significant overall preference for using the SR over the FTR (8.1 vs. 3.5, *p* < 0.001). SRs were rated as superior to FTR in all categories examined. The greatest differences between SR and FTR were in the time efficiency for creating the report via SR (8.7 vs. 2.9 pints) and the perceived great benefit of SR for inexperienced physicians in documentation through the simple and structured selection of predefined text parts (7.9 vs. 2.4 points). A detailed analysis of the user ratings is shown in Fig. 5. Individually expressed suggestions for improvement of the SR template included a greater depth of content for the elements provided.

## Discussion

The aim of this study was to evaluate the efficiency and quality of the structured creation of panendoscopy reports compared to conventional dictation. To the best of our knowledge, initial promising studies on structured digital documentation, particularly in radiology, do exist^4,5,11,13,35,36^. However, to date, there is a lack of corresponding data on the application of these methods in surgical procedures. The work to date has also focused primarily on digital documentation systems with free text input options, and simple check lists like the WHO team time out or the CLOSE acronym to improve patient safety^37,38^, while highly standardized text modules, such as those examined in this study, have hardly been researched^2,39–43^. The present study therefore aims to evaluate structured digital reporting as a potential means of improving the quality and efficiency of surgical reporting. It is intended to create a bridge to AI-supported documentation, which is currently not legally certain, fully data-protected and insufficiently quality-assured and understood^19,21^.

Therefore, a secure, legally and data protection compliant, web-based structured surgical documentation was implemented with this study, exemplified for panendoscopies. This approach makes it possible to create a standard surgical report for a standardized surgical procedure with unremarkable surgical findings with a single click. Pathological findings can be added to the structured surgical documentation with just a few clicks using a user-friendly decision tree, thus minimizing the need for free text input. The study population comprised 64 patients and 128 reports – an exceptionally large number of cases for a prospective study in the operating room, comparable to the sample sizes of retrospective studies in radiology^3–5,7,11,13,44^.

The results of our study are consistent with previous work on structured documentation in other medical specialties and show that SRs significantly improve the completeness of report content and contribute to greater user satisfaction without significant loss of time^3,26,45^.

In our study, the creation of surgical reports for panendoscopies using SR took an average of 8 seconds longer than the creation of FTR. However, this effect was not statistically significant, and the time interval only took into account the surgeons’ dictation and correction time, but not the additional transcription time required for FTR by the medical transcription service. If all the necessary steps for creating the FTR were included, the total time for creating FTRs was significantly longer and more resource-intensive than for SRs. It can be concluded from this that SR has the potential to both shorten documentation time and minimize the resource consumption for documentation, and thus, if well designed, to create time resources^46^.

Although transcription-based documentation is gradually being replaced by innovative digital systems in some healthcare systems, it remains common practice in many primary, secondary, and tertiary care settings in Europe and beyond, particularly in environments where strict data protection laws and information security requirements hinder the widespread adoption of innovative digital and AI-based systems. Nevertheless, future research should compare structured reporting not only with transcription-based free-text documentation, but also with modern digital and AI-supported reporting workflows in order to assess the relative advantages and disadvantages as well as opportunities and risks, particularly from a quality and risk management perspective.

Outside of surgical reporting, there are some studies that suggest that the introduction of SRs can also lead to an increase in documentation time^3,13,14^. This may be due, on the one hand, to the limited experience of clinical users in handling digital applications and, on the other hand, to the inadequate structure or user-friendliness of standardized digital reporting in the medical workflow. These and other individual and organizational challenges have also been confirmed in other recent studies^39,41^. Therefore, it was particularly important to the authors of the present study to implement an easy-to-use digital solution for structured reporting that could be used intuitively and quickly adapted by all clinical users. To guarantee that all relevant data can be entered, our SR template also allows free text entries if needed, despite the comprehensive structuring. It was developed in collaboration with highly experienced surgeons to ensure that it could be integrated as smoothly as possible into the clinical user’s workflow and to provide intuitive usability. The participating surgeons in this study confirmed the increase in familiarity with SR already described in the literature after just a few uses^9^.

To our knowledge, this is the first study in which a fully structured, machine-readable report template for surgical panendoscopy in head and neck oncology has been implemented and evaluated. Unlike previously published partially structured endoscopy reports, our template is based on a semantic data model and includes a visual interface for anatomical sketches, achieving a level of interoperability that has not yet been evaluated in surgical documentation. A potential limitation of our study design is the order in which the reports were produced: in all cases, the free-text report was completed before the structured report. This may have led to bias, as the documentation of cases was already practiced at the time the SR was completed. To account for this potential effect, a randomized crossover design should be considered for future studies in this context, where possible, while maintaining clinical routine and avoiding disruption to existing workflows in order to eliminate potential sequence effects and further improve methodological robustness. Furthermore, long-term compliance, user retention, and possible fatigue in report generation were not investigated in this single-center study. These aspects should be evaluated in future longitudinal studies to assess the sustainability and scalability of structured reporting tools in routine clinical practice.

This study also shows that the use of SR increases the completeness and therefore the quality of panendoscopy reports compared to FTR. These results are consistent with previous work on structured documentation outside of surgical reporting, particularly in medical imaging^4,8,27,36^. From the authors’ point of view, future studies should investigate the extent to which a slight increase in documentation time is justified if it results in a clearly structured and legible report of high quality and completeness. This could increase patient safety and promote the efficiency of subsequent treatment steps. The risk of omitting relevant findings in the report can also be reduced by pre-structuring in SR, as this ensures that all essential aspects of the documentation are covered, as it has already been shown in other medical fields^5^. In the current study, the pre-structure of the template did not include a subdivision of the summary of the panendoscopy. To provide a complete summary at the end of the report, which contains the most important pathological findings, the tumor stage, and indications for treatment options, a more detailed pre-structure should be added in future versions of the SR template or even automated text processing that provides a conclusive summary for the surgeon.

One challenge with SR is that predefined text components may limit surgeons in describing findings in their own wording and phrasing^13^. If a relevant finding is not offered as a specific option in the SR template, there is the option of selecting a similar formulation. However, this can lead to documentation that is not precise and, in the worst case, contains incorrect or misleading information. Alternatively, a general structure could be provided that can be supplemented by free text, thus allowing a high degree of flexibility for clinical users^2^. On the other hand, extensive free text entry limits the use of structured secondary data and reduces the potential for optimization in patient care and research. Therefore, a highly standardized SR template was used in this study, which covers over 90% of physiological and pathological findings, but still allows for targeted free text additions as needed.

In future studies, these effects should be examined in detail from a quality management perspective and compared with the physician-specific templates that are already in widespread use in clinical practice. Even though this was not the subject of the current study and should therefore be regarded as a limitation, the results nevertheless suggest that the establishment of national or even international standards for the medical documentation of surgical procedures should be considered in order to promote the quality and efficiency of care. Furthermore, it should be noted that the template is only consistent with national guidelines and expert consensus. As part of further development, additional, ideally international iterations should be carried out with broader expert input and aim at international harmonization. This could further promote cross-institutional applicability and standardization.

In addition, it seems sensible to integrate the SR into the system architecture of hospitals in such a way that a high level of user-friendliness is ensured. This would allow the advantages already described in the literature with regard to interoperability, standardized terminology, readability and accessibility of information to be realized more comprehensively^42,47^.

Satisfaction with the SR was higher in all categories surveyed than with the FTR. The participating surgeons stated that they preferred SRs despite the slightly higher time investment due to the associated advantages, such as the higher quality of the reports compared to the FTR. The SR template was evaluated as intuitive and easy to learn. These results are consistent with previous studies on structured documentation, which also found a preference for SR over FTR^3,9–12,27,35^. However, it should be noted that the user feedback in our study came exclusively from experienced surgeons, some of whom were also involved in the development and implementation of the SR template. This could have led to an expectation or confirmation bias, which should be considered a limitation. In addition, the perceived benefit of SR for inexperienced physicians was based solely on the subjective judgment of experienced users. This perception may not fully capture the actual usability or training effect for less experienced clinicians. Future studies should aim to involve novice users directly in the evaluation process to empirically assess the learning impact and practical utility of structured reporting in surgical education.

All surgeons were open to the use of structured digital reporting. It was therefore not possible to evaluate whether less digitally savvy participants might have reached different conclusions. The results of this single-center study with the corresponding number of cases thus form an important basis for the development of future multicenter studies with a larger number of patients. This will be necessary to further validate the current results and to evaluate the interinstitutional transferability of structured reporting in head and neck surgery.

In this study, the superiority of SR over FTR, which has already been demonstrated in other medical specialties^5,6,42,48–56^, was also confirmed for the creation of surgical reports using the example of panendoscopy reports in terms of report structuring, user satisfaction and completeness. Even though it could not be evaluated in the current study, there is further potential for SR for medical documentation, as listed below, which should be addressed more in future studies. A SR template with clickable options and decision trees can enable non-native speakers to create a grammatically and syntactically correct report, even if they have limited language skills. SR could therefore be a good way to overcome possible language barriers when creating complex surgical reports. Clinical scores, information on medications and guidelines, such as the UICC tumor stages, can be easily integrated into the user interface of the SR templates^57,58^. Such functions can support the learning process of medical personnel, especially for inexperienced doctors at the beginning of their careers^9,59^.

From a quality management perspective, it should be noted that the SR is available to all physicians involved in further treatment immediately after completion by the surgeon. This is done without delay caused by the transcription office, which usually took 1 to 2 days to transcribe the reports during the study. If information from the surgical report is needed promptly, for example in the context of emergency treatment, this can only be ensured by using SRs and creating them immediately after the diagnostic procedure or intervention^36^.

Another goal of introducing SRs is to build a database of machine-readable, structured data that can be easily used for retrospective analyses and artificial intelligence training^18,20,60,61^. This secondary data use not only improves efficiency by reducing the human resources required to collect retrospective data from unstructured paper or digital PDF documents, but also facilitates future research projects and thus helps to ensure the quality of care^44–47^.

In this study, no AI was used to create surgical reports because there are currently still significant limitations to the use of AI in medical care. These limitations arise from the legal framework for medical devices, data protection requirements, and quality management^21,22^. From the authors’ point of view, SR is a suitable bridging technology to improve the quality and efficiency of medical documentation and thus of healthcare.

In the future, AI could develop into a suitable tool for supporting structured reporting in medicine. However, to make this possible, the current challenges and insufficiently understood phenomena of AI need to be better researched and evaluated. These include the potentially unlimited number of possible inputs and outputs, the problem of erroneous or misleading outputs (so-called hallucinations), the currently limited monitoring options, restricted usability due to the enormous amounts of data and the immature legal framework^21,22^.

## Conclusion

The digitally structured medical documentation, with its increased user satisfaction compared to free text documentation, makes a valuable contribution to ensuring and improving the quality and efficiency of documenting standardized surgeries and effectively supports surgeons in creating surgical reports. Intuitive usability in everyday clinical practice and comprehensive technical integration into hospital information systems are essential to ensure the acceptance and effectiveness of SR. Furthermore, SRs provide a solid basis for the secondary use of data in medical care and research, particularly with regard to training AI models. Future developments should aim to optimize the existing infrastructure and to further investigate the possibilities of AI-supported documentation.

## Data Availability

Raw data were generated at the Department of Otorhinolaryngology – Head & Neck Surgery, University Hospital Tübingen, Tübingen, Germany and are available from the corresponding author SB on request. The data are not publicly available due to their containing information that could compromise the privacy of research participants.
